# Pharmacokinetics and Bioequivalence of Two Formulations of Apixaban Tablets: A Double-Blind, Single-Dose, Crossover Study in Healthy Subjects

**DOI:** 10.5812/ijpr-157714

**Published:** 2025-04-03

**Authors:** Erfan Abdollahizad, Azadeh Haeri, Abolghasem Jouyban, Mohammad Reza Afshar Mogaddam, Zahra Abbasian, Simin Dadashzadeh

**Affiliations:** 1 Department of Pharmaceutics and Pharmaceutical Nanotechnology, School of Pharmacy, Shahid Beheshti University of Medical Sciences, Tehran, Iran; 2 Protein Technology Research Center, Shahid Beheshti University of Medical Sciences, Tehran, Iran; 3 Pharmaceutical Analysis Research Center, Faculty of Pharmacy, Tabriz University of Medical Sciences, Tabriz, Iran; 4 Faculty of Pharmacy, Near East University, Mersin 10, Turkey; 5 Food and Drug Safety Research Center, Tabriz University of Medical Sciences, Tabriz, Iran; 6 Neurosciences Research Center, Tabriz University of Medical Sciences, Tabriz, Iran; 7 Pharmaceutical Research Center, Shahid Beheshti University of Medical Sciences, Tehran, Iran

**Keywords:** Apixaban, Bioequivalence, Pharmacokinetics, LC-MS/MS

## Abstract

**Background:**

The present study aimed to determine the pharmacokinetic parameters and bioequivalence of the test medicinal product, apixaban 5 mg tablet, and its reference product, Eliquis^®^, in healthy male and female subjects under a fasted state.

**Methods:**

Before in vivo evaluation, the quality control parameters of the products were evaluated and compared. This study was a single-dose, double-blind, 2-sequence, crossover, 2-period, randomized bioequivalence and pharmacokinetic study in 24 healthy individuals with a two-week washout period between doses. A series of blood samples were obtained over 48 hours after dose administration, and the samples were analyzed for their apixaban content using a validated liquid chromatography-tandem mass spectrometry (LC-MS/MS) technique. The pharmacokinetic parameters were computed using non-compartmental analysis.

**Results:**

Both products passed the in vitro quality control criteria. Following administration of the apixaban tablet, the area under curve (AUC)_0-t_, AUC_0-∞_, and maximum plasma concentration (C_max_) mean values for the test product were 1284.0 ng.h/mL, 1368.2 ng.h/mL, and 157.4 ng/mL, respectively, and for the reference product were 1310.6 ng.h/mL, 1406.5 ng.h/mL, and 157.6 ng/mL, respectively. The 90% confidence intervals (CI) of the geometric mean ratio for AUC_0-t _(91.4 - 105.9), AUC_0-∞_ (92.9 - 106.9), and C_max _(87.1 - 101.9) fell within the predefined accepted range of 80% - 125%. No serious adverse events were observed.

**Conclusions:**

The test product (apixaban 5 mg tablet) and reference product (Eliquis^®^) achieved regulatory requirements for bioequivalence in healthy individuals under a fasted state.

## 1. Background

Thromboembolic disorders significantly impact global health, with over 10 million people worldwide affected by venous thromboembolism annually, resulting in a case fatality rate of about 20%. Cardioembolic stroke, another thromboembolic disorder and a severe complication of atrial fibrillation, affects approximately 33 million individuals globally. Anticoagulants are the first line of prevention or treatment for thromboembolic diseases (1, 2). Traditional anticoagulants such as heparin and warfarin present challenges and limitations. Warfarin, as an oral anticoagulant, has an unfixed anticoagulation effect, interacts with a broad range of drugs and foods, and requires routine monitoring, complicating clinical practice. In response to these limitations, direct oral anticoagulants (DOAs) were introduced to the pharmaceutical market. The DOAs have fixed effect profiles without the need for routine monitoring (3, 4).

Apixaban is a DOA that targets factor Xa in the coagulation cascade. It reversibly inhibits factor Xa coupled to platelets or inactivated Xa in the prothrombinase complex as the free factor Xa. Apixaban is prescribed for venous thromboembolism prophylaxis in patients prone to blood coagulation, such as those hospitalized with acute illnesses or following major orthopedic surgery, as a stroke preventer in atrial fibrillation patients, or as a treatment for venous thrombosis. The recommended dose of apixaban is typically 2.5 to 10 mg twice a day (5, 6).

Apixaban is available as immediate-release tablet formulations. It is primarily absorbed in the small intestine, and its absorption decreases as the drug molecules travel along the intestinal lumen. The oral bioavailability of apixaban tablets is about 50%. The maximum plasma concentration (C_max_) is reached approximately 3 hours after oral administration (7). Following oral administration of apixaban with food, drug exposure — i.e., area under curve (AUC) the plasma concentration-time and C_max_ — was comparable to that of the fasting state (8). In the study by Song et al. (9), administration of the whole tablet with food reduced C_max_ and AUC by 15% and 20%, respectively; however, these findings were not considered clinically significant. These researchers also investigated the bioavailability of apixaban from the crushed tablet. According to the results, the crushed tablet exhibited a decrease in apixaban exposure compared to the whole tablet (21% and 16% reduction in C_max_ and AUC, respectively), but it was not clinically significant (9). It can be concluded that administration of apixaban with food or as a crushed tablet has no significant effect on its efficacy. Therefore, patients with swallowing difficulties can administer the tablet by crushing and dispersing it in water.

With a relatively low volume of distribution (0.3 L/kg), apixaban is primarily found in extracellular fluids and has a protein binding of 93%, with the highest affinity for albumin. Apixaban has a total plasma and renal clearance of 3.3 L/h and 0.9 L/h, respectively (the renal to total clearance ratio of the absorbed drug is about 27%). The elimination half-life (t_1/2_) of apixaban is about 12 hours, and its elimination from the body occurs via various pathways, including metabolism [mainly by cytochrome P450 (CYP) 3A4] and excretion of the unmodified drug through the biliary and renal systems. With decreased renal function, t_1/2_ and AUC increased; however, mild to moderate hepatic impairment had no discernible effect on the pharmacokinetic profile. Apixaban is not recommended for patients with end-stage chronic kidney disease or severe hepatic impairment. Apixaban is a substrate of both P-glycoprotein and CYP 3A4, and therefore its concurrent administration with powerful inducers or inhibitors of CYP 3A4 and the efflux carrier should be undertaken with caution (7, 10). Further intrinsic factors such as age, sex, body weight, and race can slightly affect the pharmacokinetic parameters; however, clinical trials confirm that no dose adjustment of apixaban is needed in these situations (10).

Bioequivalence studies are crucial for the development of generic drug products. The target of such studies is to evaluate the therapeutic compatibility of the brand/original and the generic products when administered at the identical molar dose of the active component under similar conditions. The basis of the bioequivalence assessment is the concept that a product's therapeutic profile is directly correlated with the active component’s concentration in the circulating blood. As a result, two therapeutic products are bioequivalent if their concentration-time profiles are similar enough to confer comparable clinical efficacy (11, 12). Therefore, a comparison of the extent and rate of drug absorption following the administration of pharmaceutical equivalent products is essential to ensure the achievement of equal efficacy.

## 2. Objectives

Due to the clinical importance of the apixaban tablet and the necessity of bioequivalence for generics, the current study was undertaken to evaluate the pharmacokinetic parameters and determine the bioequivalence of a generic product of apixaban 5 mg tablet (test) with a relevant reference product (Eliquis^®^ 5 mg tablet) in healthy Iranian subjects under fasting condition.

## 3. Methods

### 3.1. In Vitro Quality Control Tests

#### 3.1.1. Content Assay

The content assay was performed according to previous reports (13, 14). In detail, 10 tablets from each product were weighed. After powdering the tablets, the equivalent of the average weight of the tablets was separated from the mixed powder and dissolved in methanol (50 mL). After vortexing for 0.5 hours and filtration, 10 μL of the solution was injected into the high-performance liquid chromatography (HPLC). Then, a solution with a concentration of 100 μg/mL of reference apixaban hydrochloride was prepared in methanol. Next, 10 μL of the filtered standard solution was injected separately into the HPLC. The content assay of the products was calculated based on the following equation. Acceptance criteria for the content assay are 90.0% - 110.0%: Assay% = (r_u_/r_s_) × (C_S_/C_U_) × (M_r1_/M_r2_) × 100, where r_u_ and r_s_ are peak area responses of the drug sample and the drug standard solution, respectively. C_S_ and C_U_ are the concentration of the drug standard solution and the drug sample solution's nominal concentration, respectively. M_r1_ and M_r2 _are the mean weight of the batch (mg) and mg of weighed apixaban, respectively.

#### 3.1.2. Content Uniformity

To perform the content uniformity test, 10 tablets were randomly selected from each of the reference and test products. After determining the content of each tablet separately according to the method mentioned in the previous section, the acceptance value was calculated according to the following equation, and a value lower than 15 is acceptable: Acceptance value =│M - $\overset{-}{X}$│+ ks, which M,$\;\overset{-}{X}$ and s are the reference value, mean of individual contents, and sample standard deviation, respectively. K is the acceptability constant which is 2.4 for n = 10 (15).

#### 3.1.3. In Vitro Dissolution Testing

The 0.05 M phosphate buffer containing 0.05% sodium lauryl sulfate (pH = 6.8), with a volume of 900 mL, was used as the dissolution medium. Apparatus II was used for this purpose. The rotation speed of the paddles was 75 rpm, and the temperature was fixed at 37°C. At 5, 10, 20, 30, and 45 minutes, 5 mL of the dissolution medium was sampled (sample solution) and replaced by the same volume of fresh dissolution medium (13, 16). The sample solutions were filtered, and their drug concentration was determined with HPLC. At least 80% of apixaban must be dissolved within 45 minutes.

#### 3.1.4. Analytical Method for In Vitro Tests

To measure the amount of drug in the content assay, content uniformity, and dissolution test, the HPLC method with a UV detector was used. The measurement was performed using an Azura solvent delivery pump (Knauer, Germany), Azura UV detector (Knauer, Germany), a C18 LiChrosorb-100 RP 18 column (250 mm × 4.6 mm, 5 µm, Knauer, Germany) at 35°C, and with a gradient elution of methanol and KH_2_PO_4_ solution (0.025 mM, pH = 3 adjusted by H_3_PO_4_) with a 1.0 mL/min flow rate and detection at 220 nm. Initially, the V/V proportion of methanol to KH_2_PO_4_ solution was 50:50; then at 9 minutes, it was 80:20. At 12 minutes, the proportion changed to 50:50 and did not change until the end of the run at 15 minutes. The calibration curve at the measurement range (1 - 6 μg/mL) was linear (R^2^ = 0.998). The accuracy and precision of the utilized technique were within an acceptable range.

### 3.2. Study Population

Adult healthy men and women who passed medical history, physical exams, and clinical laboratory testing (blood coagulation, serology, hematology, blood chemistry, and urinalysis tests) were eligible to participate in the study. Participants (24 individuals) were between 18 and 47 years old. For both genders, the Body Mass Index (BMI) ranged from 19 to 30 kg/m^2^ with a minimum weight of 50 kg. Female participants had a pregnancy test performed before the research began, and all sexually active individuals were required to utilize effective contraception methods until one month following the initial dosage. Subjects taking any medication within two weeks prior to the initial dosage were excluded. Active smokers or subjects with any history of allergy, active hemorrhagic or coagulation diseases, hematologic abnormalities, and any other acute or chronic disease were also excluded. All participants were informed of the study’s methodology and conditions, including rights, obligations, and potential adverse effects, and written informed consent was obtained.

### 3.3. Study Design

This single-dose, randomized, double-blind, crossover, two-period, two-sequence study was conducted at the Bioequivalence Study Center, Shahid Beheshti University of Medical Sciences, Tehran, Iran to assess the pharmacokinetic features and the bioequivalence of the reference tablet (Eliquis^®^; 5 mg apixaban; Lot No. EK3084; manufactured by Pfizer, Turkey) and test tablet (5 mg apixaban; lot No. 214104; manufactured by Zist Arvand Pharmed, Iran). Ethical principles for human subject research were based on the Declaration of Helsinki (17) and the International Conference on Harmonization’s (ICH) guidelines for good clinical practice (18), and the study was approved by the Ethics Committee of Shahid Beheshti University of Medical Sciences (IR.SBMU.PHARMACY.REC.1400.138).

### 3.4. Drug Administration and Blood Sampling

All participants were randomly assigned to take either the reference or test product, with a two-week washout period. Each participant fasted overnight (10 hours) before taking the medicine with 240 mL of water; they were deprived of water and food for 4 hours after taking the dose, and their diets were standardized on study days. The on-site medical team monitored the subjects’ health conditions and vital signs during the administration and sample times and followed up with them throughout the study. Blood samples (5 mL) from each subject were collected in heparinized tubes at 0 (pre-dose), 0.5, 1, 1.5, 2, 2.5, 3, 3.5, 4, 5, 6, 8, 10, 12, 24, 32, and 48 hours following drug administration. Blood samples were centrifuged at 4000 rpm for 10 minutes, and the plasma of the blood samples was collected and stored at -80°C until the analysis day.

### 3.5. Bioanalytical Method Development and Validation

Apixaban plasma concentration was determined by applying a validated LC-MS/MS technique involving dabigatran etexilate as the internal standard (IS). The accuracy, precision, and linearity of the analytical method were evaluated during validation to verify its reliability (19). The following is a condensed description of the bioanalytical method: To isolate apixaban from human plasma samples, a protein precipitation process was utilized. For this purpose, 250 μL of plasma was transferred to a microtube, and 20 μL of 0.25 mg/L IS solution was added. Then, 250 μL acetonitrile was added to the mixture to precipitate the protein. After centrifugation at 10000 rpm for 5 minutes, the clear supernatant solution (20 μL) was injected into the chromatographic system. The following chromatographic conditions were established: The column was Agilent C18 (60 × 4.6 mm, 5 µm), flow rate and column temperature were 0.6 mL/min and 35°C, respectively, and the mobile phase was a mixture of methanol and water (65:35, v/v). Apixaban and the IS were detected by a triple-quadrupole mass spectrometer Quattro Micro (Waters-Micromass, UK) in positive-mode electrospray ionization in multiple-reaction-monitoring (MRM) mode (m/z 460.1 → 199.2 for apixaban and m/z 628.2 → 289 for IS). Mass Lynx 4.1 was used to compute peak areas for apixaban and IS, and the content concentration of the sample was determined by the peak area ratio based on the calibration curve.

### 3.6. Pharmacokinetic and Statistical Analysis

The standard noncompartmental method by PKSolver software (20) was utilized to compute the pharmacokinetic parameters of apixaban in plasma. For each volunteer, the plasma concentration-time curve was directly observed to determine the C_max_ and the time to reach it (T_max_). The log-transformed plasma concentration-time curve’s terminal segment slope was used to derive the elimination rate constant (K), and t_1/2_ was calculated as 0.693 × K^-1^. AUC_0-t _(the AUC from time zero to the last time of the quantifiable concentration) was determined by applying the linear trapezoidal method, and AUC_0-∞ _as the extrapolated AUC from time zero to infinity was obtained by the following equation: AUC_0-∞_ = AUC_0-t _+ C_t_ × K^-1^, where C_t_ represents the last measurable concentration.

The study utilized a linear analysis of variance model (ANOVA) to establish the 90.0% CI for the geometric mean ratio of pharmacokinetic values (AUC_0-t_, AUC_0-∞_, and C_max_) after log transformation. The statistical findings informed the conclusion of bioequivalence between the reference product and the test. If the calculated 90% CI around the ratio of geometric means of pharmacokinetic values fell entirely within the bioequivalence ranges of 80% to 125%, it concluded that the test product demonstrated bioequivalence to the reference product (21, 22). T_max_ was analyzed by the Wilcoxon signed-rank test. A significance level of P < 0.05 was considered to indicate statistical significance.

## 4. Results

### 4.1. In Vitro Quality Control of the Products

**Figure 1.**
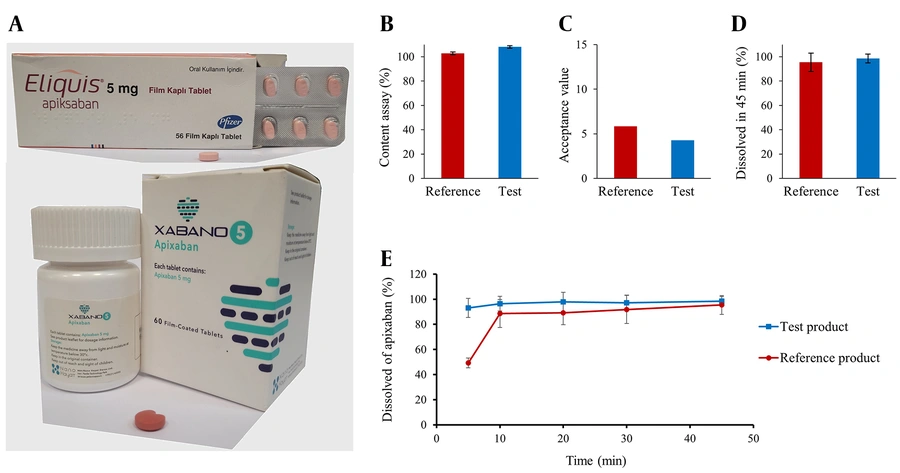
In vitro quality control assessment of the reference and the test product: A, the picture of the products; B, the result of the content assay (n = 3); C, content uniformity (n = 10); D, dissolution test (n = 12); E, dissolution profiles at different time points (n = 12)

Before evaluating the in vivo bioequivalence, the quality of the products in terms of content assay, content uniformity, and dissolution rate was investigated. The results of the in vitro quality control tests are summarized in Figure 1. 

### 4.2. Subject Demographic Characteristics

**Figure 2.**
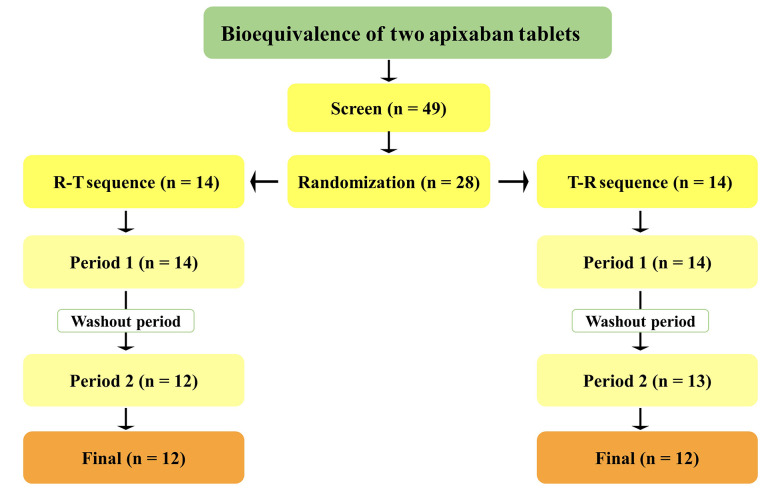
Study design and subjects’ disposition in the present bioequivalence study

The study was conducted on 24 participants (Figure 2), and Table 1 summarizes their demographic information.

**Table 1.** The Study Subjects’ Demographic Characteristics

Parameters	Mean ± SD	Range
**Male/female **	14/10	-
**Age (y)**	31.8 ± 9.3	18 - 48
**Height (cm)**	172.4 ± 8.8	150 - 186
**Weight (kg)**	71.9 ± 14.3	50 - 98
**BMI (kg/m** ^**2**^ **)**	24.0 ± 3.7	19.4 - 30.2

Abbreviation: BMI, Body Mass Index.

### 4.3. Bioanalysis and Method Validation

An LC-MS/MS technique was utilized to measure the concentration of apixaban in plasma samples. Under the described chromatographic conditions, no endogenous interference was observed in the blank plasma samples from six different sources at the retention times for apixaban (2.6 minutes) or the IS (3.6 minutes). The linearity of the plasma calibration curve was observed within the concentration range of 4.0 - 300.0 ng/mL with Y = 0.0538X - 0.1298 as the regression equation (R^2^ = 0.991). The average recovery of apixaban was 98 ± 4%, and the limit of quantification (LOQ) was 4.0 ng/mL. Apixaban plasma samples were stable for at least one month at -80°C. The intra- and inter-day accuracies and precisions of the analysis method, calculated respectively as the percentage of the relative error (RE%) and percentage of coefficient of variation (CV%), were less than 15% in accordance with guidelines (19).

### 4.4. Pharmacokinetic Parameters

**Figure 3.**
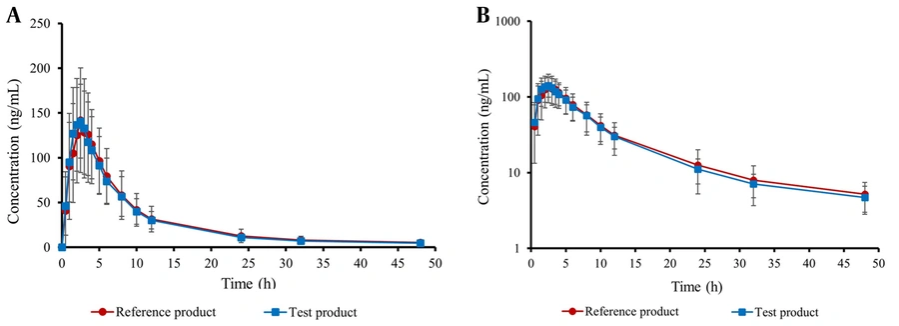
A, mean plasma concentration-time curve; and B, semi-logarithmic plasma concentration-time curve of the test and the reference product

Table 2 provides an overview of the key pharmacokinetic characteristics of both test and reference apixaban products, including AUC_0-t_, AUC_0-∞_, C_max_, T_max_, t_1/2_, and K. Moreover, the curves illustrating the mean plasma concentration-time and semi-logarithmic curves of the drug following test or reference product administration are shown in Figure 3. There was no significant difference in AUC_0-t_, AUC_0-∞_, and C_max_ between the test product and the reference product (P > 0.05).

**Table 2.** The Pharmacokinetic Parameters of Apixaban Following Administration of the Test and Reference Products to 24 Healthy Subjects

Pharmacokinetic Parameter	Reference Product		Test Product	
	Mean ± SEM	Geometric Mean	Mean ± SEM	Geometric Mean
**AUC**_**0-t**_ ** (ng.h/mL)**	1310.6 ± 101.2	1222.9	1284.0 ± 85.1	1207.2
**AUC**_**0-∞**_ ** (ng.h/mL)**	1406.5 ± 105.2	1319.8	1368.2 ± 88.7	1290.6
**C**_**max**_ ** (ng/mL)**	157.6 ± 12.5	145.7	157.4 ± 10.1	149.8
**T**_**max**_^**a**^ ** (h)**	2.5 (1 - 3.5)	-	2.5 (1.5 - 4)	-
**K (1/h)**	0.075 ± 0.005	0.069	0.072 ± 0.004	0.069
**t**_**1/2 **_ **(h)**	11.1 ± 1.4	9.95	10.4 ± 0.6	10.0

Abbreviations: AUC, area under curve; C_max_, maximum plasma concentration; T_max_, time to reach C_max_; t_1/2_, elimination half-life.

^a^ Values of T_max_ are shown as median (range).

### 4.5. Bioequivalence of the Generic Product

The 90% CI for the geometric mean ratio of log-transformed C_max_, AUC_0-t, _and AUC_0-∞_ of the test product to the reference product were 87.1 - 101.9, 91.4 - 105.9, and 92.9 - 106.9, respectively. The results fell within the permitted 80% - 125% range (21, 22), indicating that the test product and the reference product are bioequivalent (Table 3). Additionally, Wilcoxon’s non-parametric test for T_max_ showed no significant difference between the two formulations (P = 0.923).

**Table 3.** Bioequivalence Analysis of Maximum Plasma Concentration and Area Under Curve and Nonparametric Test of Time to Reach Maximum Plasma Concentration Between the Reference And Test Apixaban Tablets in Healthy Subjects

Parameters	T/R GMR (%)	90% CI (%)
**C**_**max**_ ** (ng/mL)**	94.2	87.1 - 101.9
**AUC**_**0-t**_ ** (ng.h/mL)**	98.4	91.4 - 105.9
**AUC**_**0-∞**_ ** (ng.h/mL)**	99.8	92.9 - 106.9
	**Reference**	**Test**
**T**_**max**_^**a**^ ** (h)**	2.43 ± 0.10	2.41 ± 0.14

Abbreviations: T/R GMR, geometric mean ratio of (reference/test); 90% CI, 90% confidence intervals; C_max_, maximum plasma concentration; AUC, area under curve; T_max_, time to reach C_max_.

^a^ Values of T_max_ are shown as mean ± SEM.

## 5. Discussion

Reducing healthcare costs is a concern for numerous governments worldwide, and the creation of generic versions of current pharmaceuticals could lead to substantial cost savings. When a patent of a drug product expires, generic versions of the drug can be sold at a substantially lower price because their manufacturers are spared the cost of initial registration trials (23). Since the therapeutic responses of products containing similar doses of medicine may vary significantly, these disparities are generally well associated with disparities in plasma drug levels caused primarily by changes in the absorption profile. Therefore, generic medications need to pass a bioequivalence study to verify therapeutic equivalence with brand-name drugs (12, 24).

Apixaban, one of the DOAs, is frequently prescribed for the prevention or treatment of thromboembolic disease (25). This study aimed to investigate the bioequivalence between two 5 mg tablet formulations of apixaban in healthy Iranian subjects. To achieve this objective, after studying and confirming the in vitro quality control of the products, a double-blind, two-period, two-sequence crossover trial was designed, and 24 healthy males and females participated in it. No serious adverse events were observed, and none of the observed events adversely affected the subjects’ safety.

Upon bioanalysis of the plasma samples using a validated LC-MS/MS method and pharmacokinetic analysis by a non-compartmental method, the values of major pharmacokinetic parameters including AUC_0-t_, AUC_0-∞_, t_1/2_, C_max_, T_max_, and K were obtained for both test and reference products. The obtained geometric mean of C_max_, AUC_0-t_, and AUC_0-∞_ for test and reference formulations were 149.8 vs. 145.7 ng/mL, 1207.2 vs. 1222.9 ng·h/mL, and 1290.6 vs. 1319.8 ng·h/mL, respectively.

The mean pharmacokinetic parameter values observed in our study are similar to those reported in the literature (8, 9, 26, 27). For example, Song et al. (9) studied the pharmacokinetic parameters of apixaban following oral administration of 5 mg tablets to 22 healthy subjects under fasting condition. They reported that respective mean values for C_max_, T_max_, AUC_0-∞_, and t_1/2_ were 121.3 ng/mL, 3 hours, 1229 ng·h/mL, and 9.87 hours, which are comparable to our study. Wang et al. (26), in a study on apixaban’s pharmacokinetic differences between healthy and end-stage renal disease subjects, found that mean values for C_max_, T_max_, and AUC_0-∞_ in the healthy subjects who received a 5 mg apixaban tablet were 126 ng/mL, 2 hours, and 1265 ng·h/mL, respectively. Two other studies have also found similar results (8, 27).

No significant difference was observed in the parameters between the two formulations. In addition, the 90% CIs for the log-transformed test/reference ratios of AUC_0-t_, AUC_0-∞_, and C_max_ were within the accepted bioequivalence range of 80% - 125%. These results indicate that apixaban 5 mg tablets manufactured by Zist Arvand Pharmed (Iran) and Pfizer (Turkey) are bioequivalent in healthy human subjects, and the two formulations can be used interchangeably in patients.

### 5.1. Conclusions

This study demonstrated that the generic 5 mg apixaban tablet (Zist Arvand Pharmed, Iran) is bioequivalent to the reference 5 mg apixaban tablet (Eliquis^®^, Pfizer) when administered as a single dose in healthy Iranian subjects under fasting condition.

## Data Availability

The datasets collected or analyzed in the current investigation are accessible upon reasonable request from the corresponding author.
